# Hydrostatic pressure-dependent changes in cyclic AMP signaling in optic nerve head astrocytes from Caucasian and African American donors

**Published:** 2009-08-20

**Authors:** Lin Chen, Thomas J. Lukas, M. Rosario Hernandez

**Affiliations:** 1 Department of Ophthalmology Northwestern University, Feinberg School of Medicine, Chicago, IL; 2 Molecular Pharmacology, Northwestern University, Feinberg School of Medicine, Chicago, IL

## Abstract

**Purpose:**

Investigate the effect of hydrostatic pressure (HP) on 3′, 5′-cyclic adenosine monophosphate (cAMP) levels and downstream signaling in cultures of normal optic nerve head (ONH) astrocytes from Caucasian American (CA) and African American (AA) donors.

**Methods:**

Intracellular cAMP levels were assayed after exposing ONH astrocytes to HP for varying times. Quantitative RT–PCR was used to determine the expression levels of selected cAMP pathway genes in human ONH astrocytes after HP treatment. Western blots were used to measure changes in the phosphorylation state of cAMP response element binding protein (CREB) in astrocytes subjected to HP, ATP, and phosphodiesterase or kinase inhibitors.

**Results:**

The basal intracellular cAMP level is similar among AA and CA astrocytes. After exposure to HP for 15 min and 30 min in the presence of a phosphodiesterase inhibitor a further increase of intracellular cAMP was observed in AA astrocytes, but not in CA astrocytes. Consistent with activation of the cAMP-dependent protein kinase pathway, CREB phosphorylation (Ser-133) was increased to a greater extent in AA than in CA astrocytes after 3 h of HP. Exposure to elevated HP for 3–6 h differentially altered the expression levels of selected cAMP pathway genes (ADCY3, ADCY9, PTHLH, PDE7B) in AA compared to CA astrocytes. Treatment with ATP increased more CREB phosphorylation in CA than in AA astrocytes, suggesting differential Ca^2+^ signaling in these populations.

**Conclusions:**

Activation of the cAMP-dependent signaling pathway by pressure may be an important contributor to increased susceptibility to elevated intraocular pressure and glaucoma in AA, a population at higher risk for the disease.

## Introduction

Astrocytes are the predominant glial cell type in the mammalian brain and are essential for neuronal development, neuronal activity, and regulation of localized inflammatory responses. In the nonmyelinated optic nerve head (ONH) astrocytes play an important role at providing metabolic and structural support to the axons, forming the interface between connective tissue surfaces and surrounding blood vessels. In human glaucoma and in experimental glaucoma in monkeys, the astrocytes of the optic nerve respond to the compression of the lamina cribrosa with changes in protein expression and morphology [1]. Many studies have suggested that mechanical stress created by elevated hydrostatic pressure can influence the responses of the ONH astrocytes in vitro [2-4]. The signaling pathways by which mechanical forces modulate astrocytes function remain largely unknown.

In the current study, we focused on 3′5’-cyclic adenosine monophosphate (cAMP), a second messenger that regulates important cellular functions, including proliferation, differentiation, and apoptosis. cAMP is generated from intracellular ATP by membrane-associated adenylate cyclases after activation by various receptors coupled to G-proteins. There is also recent evidence of changes in selected cAMP signaling genes in glaucomatous astrocytes [5]. Because of known effects of pressure on ONH astrocytes [6-10], we determined the effects of hydrostatic pressure (HP) on cAMP levels in normal ONH astrocytes from age-matched Caucasian American (CA) and African American (AA) normal donors without history of eye disease or glaucoma. We also determined the effects of HP on cAMP response element binding protein (CREB), a transcription factor activated by cAMP-dependent protein kinase phosphorylation at serine 133 [11]. Hormones, such as glucagon, parathydroid hormone, or epinephrine, can activate CREB through the cAMP- protein kinase (PKA) signaling pathway [11]. Complicating the investigation of CREB phosphorylation is the fact that PKA is not the only protein kinase that is responsible for phosphorylation and activation of CREB. Other protein kinase pathways including Ca^2+^-dependent protein kinases and extracellular signal regulating kinase (ERK) can also lead to CREB phosphorylation [12].

The results of our studies suggest that there is differential regulation of cAMP-mediated signaling in populations of ONH astrocytes, especially in their response to pressure. AA astrocytes are more sensitive to pressure than CA astrocytes with respect to cAMP-mediated signaling, while CA astrocytes are more sensitive to extracellular ATP-mediated signaling. Thus, certain populations of ONH astrocytes may be more susceptible to glaucoma-inducing conditions such as elevated intraocular pressure.

## Methods

### Astrocyte culture

Normal human eyes from donors with no history of chronic central nervous system or eye disease were obtained from the National Disease Research Interchange (NDRI) within 2–4 h of death. Optic nerves were dissected and processed within 24 h of death to generate ONH astrocyte cultures. Cultures were derived from twenty three donors. Eleven were AA donors, 8 male, 3 female, aged 38–74 years. Twelve were CA donors, 9 male, 3 female, ages 29–68 years of age. Primary cultures of human ONH astrocytes were purified, characterized, and maintained as previously described [13]. Briefly, primary cells grown from human optic nerve head explants were cultured for 3–4 weeks. To select astrocytes by immunopanning, cell suspensions were placed on a P100 panning dish coated with C5 anti-neuroepithelial antibody and allowed to attach for 30 min. Nonadherent cells were plated on a second dish coated with anti-Thy1.1 antibody to deplete microglia and meningeal cells. Finally, remnant nonadherent cells were plated on a noncoated dish. Purified cells were immunostained with astrocyte markers: glial gibrillary acidic protein (GFAP), vimentin, Pax2, A2B5, nestin, and neural cell adhesion molecule (NCAM). Other cell types were characterized by HLA-DR for microglia and smooth muscle actin for vascular smooth muscle. The proportion of GFAP+ astrocytes in the cultures was determined by flow cytometry. About 95% of the cells that adhered to the C5 dish were GFAP+ astrocytes. GFAP+ astrocytes expressed vimentin, Pax2, nestin and NCAM, but not A2B5. From the Thy1.1 dish, 60%–75% cells were GFAP+ astrocytes and the remainder cells were GFAP- cells. Using cloning rings, we eliminated fibroblast-like cells, smooth muscle and meningeal cells from astrocyte cultures.ONH astrocytes were cultured at 37 °C in Dulbecco’s modified Eagle’s medium (DMEM) and F-12 with 5% fetal bovine serum (FBS). The media also contained 5 μl/ml of an antibiotic mixture containing 10,000 U/ml penicillin, 10 mg/ml streptomycin, and 25 μg/ml amphotericin B (30–004-CL; CellGrow, Herndon, VA). The astrocytes were cultured in a humidified atmosphere of 95% air and 5% CO_2_. All experiments used astrocytes at the third to fifth passages with 5% FBS DMEM-F-12.

### Application of elevated hydrostatic pressure

Cells were grown to confluence in six-well plates then were transferred to HP chambers equipped with a manometer. Plates were placed on a supporting rack in the chamber that had a water reservoir below the rack to maintain 98% humidity. Elevated HP was created by compression of the gas phase in the closed chamber, as described previously [2,7]. Briefly, pressure was raised to 60 mmHg (8.6 kPa) above ambient pressure by filling the chambers with a mixture of 92% air and 8% carbon dioxide. The elevated CO_2_ concentration helped to maintain a 7.3–7.4 pH [14]. Partial oxygen pressure (pO_2_) was measured using a micro dissolved oxygen electrode, DO-166MT (Lazar laboratories, Los Angeles, CA) [2,15]. Once the sealed chambers were filled, cells were placed in a tissue culture incubator and maintained at 37 °C for the desired treatment time (15 min to 6 h). A pressure gauge was used to monitor the chamber pressure, which did not change over the course of the experiment. Control groups were cultured at ambient pressure at the same time.

### RNA extraction, cDNA synthesis, and real time PCR

Total RNAs were extracted from human astrocytes using Trizol reagent (Invitrogen, Carlsbad, CA) following manufacturer’s manual. As recommended by the manufacturer, 1 μg of total RNA was used for first-strand cDNA synthesis. Real-time RT–PCR was performed using SYBR Green on Bio-Rad Real-Time PCR System (MyIQ; Bio-Rad Laboratories, Hercules, CA). The final reaction mixture contained 10 ng of cDNA, 100 nM of each primer, 10 μl of 2X SYBR® Green PCR Master Mix (Bio-Rad Laboratories) and RNAase free water to complete the reaction mixture volume to 20 μl. All reactions were performed in triplicate. PCR was performed with a hot-start denaturation step at 95 °C for 10 min, and then was performed for 40 cycles at 95 °C for 15 s and 60 °C for 1 min. Fluorescence was read during the reaction, which enabled continuous monitoring of the amount of PCR product. Changes in cycle time (ΔC_t_) were calculated based upon 18S as well as *RPL13A* mRNA. Quantitation of gene expression was done the using standard curve method. cDNA from all samples were mixed and used as standards. Serial dilutions (1:5, 1:10, 1:40, 1:160, and 1:640) of the mixed cDNA were used to generate standard curves. The sequences of primers used in real-time PCR are shown in Table 1.

**Table 1:** Primers used for quantitative RT–PCR of selected genes in human ONH astrocytes

**Gene**	**GenBank accession number**	**Primer**	**Sequences (5′ to 3′)**	**Size**
*ADCY3*	AF033861	F	TTGACTGCTACGTGGTGGTCATGT	114
		R	TGCAGAGCACGAAGAGGATGATGT	
*ADCY9*	AF036927	F	TCTCCTGCTCTTGTTGGTCTGGTT	147
		R	ATGATGTTCCTCAGCAGCCAGTCT	
*CAP2*	NM_006366.2	F	ATGGACAGTATGGTGGCCGAGTTT	94
		R	CCTGGAAAGCACTGTGCACCATTT	
*PTHLH*	NM_002820	F	CGACGACACACGCACGCACTTGAAACTT	118
		R	AACCAGTCTCCGCTGCATCGTCT	
*PDE7B*	NM_018945	F	AGGATGCACAGGACAGGCACTTTA	88
		R	ACAGACCCTTTCACTCCACTGCTT	
*18S*	NR_003286	F	TCTAGATAACCTCGGGCCGA	91
		R	ACGGCGACTACCATCGAAAG	
*RPL13A*	NM_012423	F	TTAATTCCTCATGCGTTGCCTGCC	122
		R	TTCCTTGCTCCCAGCTTCCTATGT	

### cAMP assay

Primary cultures ONH astrocytes obtained from 8 AA donors and 8 CA donors were grown in 60 mm dishes until 80% confluence. Cells were then conditioned for 18 h in serum-free media that contained DMEM-F12 along with insulin-transferrin-selenous acid supplement (ITS; BD Biosciences, San Jose, CA). For each donor culture, cells were divided into four groups: basal serum-free conditions; 500 µM 3-isobutyl-1-methylxanthine (IBMX), a phosphodiesterase inhibitor to stabilize cAMP, 500 µM IBMX plus exposure to 60 mmHg HP for 15 min and for 30 min, respectively. Cells were washed with ice-cold PBS. They were then lysed in 95% chilled ethanol for 1 h and centrifuged at 2,000x g for 15 min at 4 °C. The supernatant was allowed to evaporate via a SpeedVac and cells were resuspended in 100 μl of assay buffer. Intracellular cAMP was measured using the cAMP Biotrak Enzyme Immunoassay Kit (RPN225; Amersham Biosciences, Piscataway, NJ) in accordance with the instruction manual. Standards and samples were added to a microtiter plate that had been coated with donkey anti-rabbit IgG. Sodium acetate buffer was added followed by a solution containing cAMP rabbit polyclonal antibody. Plates were incubated at 4 °C for 2 h on a plate shaker. A solution containing horseradish peroxidase conjugated to cAMP was added, and the mixture was allowed to incubate at 4 °C for 1 h. Following this incubation, the plates were washed with phosphate buffer and then dried. Enzyme substrate containing tetramethylbenzidine was added to each well, and the cells were incubated at room temperature for 1 h after which time the reaction was terminated by 1 M sulfuric acid. The plate was read immediately at an optical density of 450 nm using a plate reader. Protein concentration of each sample was determined from the ethanol-insoluble pellet using the BCA reagent (Pierce, Rockford, IL). cAMP concentration per well was expressed as cAMP pmol per mg of protein and used to calculate the fold change relative to basal control value. Each value represents the mean fold change (±SEM) of eight independent experiments using 8 AA and 8 CA astrocyte cultures performed in triplicate.

### Preparation of nuclear extract and western blot analysis

Cells were plated at 30,000 cells per well and grown to confluence. Cells were then conditioned in serum free media (DMEM-F12+ITS supplement) for 18 h, then exposed to HP in the presence and absence of PKA inhibitor N-[2-(p-bromocinnamylamino)ethyl]-5-isoquinolinesulfonamide dihydrochloride (H-89; Sigma-Aldrich, St. Louis, MO). Some experiments were also done in the presence of IBMX. Nuclear proteins were extracted by using Nuclear Extract kit (#40010; Active Motif, Carlsbad, CA). Briefly, cells were removed using a cell scraper and centrifuged at 500× g for 5 min at 4 °C. The cells were treated with 300 μl hypotonic buffer and allowed to swell before they were incubated on ice for 15 min. Next, 25 μl of extraction buffer were then added and the sample vortexed to disrupt cell membranes. The mixture was centrifuged for 30 s at 14,000× g at 4 °C. Pellets containing crude nuclei were resuspended in 50 μl complete lysis buffer, vortexed for 10 s at highest setting, then incubated for 30 min on ice on a rocking platform set at 150 rpm, and centrifuged again at 14,000× *g* for 10 mins. The supernatants containing the nuclear extracts were collected and stored at −80 °C until required. Nuclear extract protein (5 μg) from each sample was fractionated by sodium dodecyl sulfate PAGE on NuPAGE 10% Bis-Tris gels (Invitrogen). Electrophoresed proteins were then transferred to PVDF membranes (Millipore, Billerica, MA) in an XCell Blot apparatus (Invitrogen). The membranes were subsequently incubated with 1:1,000 primary antibody anti–phospho-CREB pSer-133 (#9198; Cell Signaling Technology, Danvers, MA) and anti-total-CREB (#9197; Cell Signaling Technology) in 5% w/v BSA in Tris-buffered saline (20 mM Tris, 140 mM NaCl, pH 7.4) containing 0.1% v/v Tween-20 (Calbiochem, San Diego, CA). Membranes were incubated at 4 °C with gentle shaking overnight and then with 1:3,000 horseradish peroxidase-conjugated anti-rabbit secondary antibodies (# 7074; Cell Signaling Technology) for 1 h at room temperature. Finally, the membranes were developed using an ECL kit (BM Chemiluminescence; Roche, Indianapolis, IN).

### Imaging of Ca^2+^ transients in ONH astrocytes

Subconfluent monolayers of astrocytes were loaded for 20 min with 10 µM fluo-4 AM (Molecular Probes, Eugene, OR) in DMEM serum-free medium at room temperature. They were then washed with Krebs-Ringer solution buffered with KRH media, which contained 125 mM NaCl, 5 mM KCl, 1.2 mM MgSO_4_, 2 mM CaCl_2_, 10 mM glucose, and 25 mM HEPES-NaOH, pH 7.4. Afterwards, they were incubated in KRH medium for 30 min. Cells were then mounted on the stage of a confocal-scanning microscope (Zeiss LSM 510 Meta; Zeiss, Gottingen, Germany; Objective w.i. 40X). Ca^2+^ transients were elicited by adding 1 mM ATP. Fluorescence was recorded for 5 min from up to three cells simultaneously by scanning each cell with the 488 nm line of an argon laser every 1.54 ms. Baseline fluorescence was collected for 15–20 s before addition of ATP. Peak areas were determined from baseline-subtracted data using Graph Pad Prism 4 (Graph Pad Software, San Diego, CA).

### ATP stimulation of CREB phosphorylation in ONH astrocytes

ONH astrocytes were cultured in six well plates to 80% confluence and conditioned in serum free media as described in the previous section. ATP was added to a final concentration of 100 μM and cells incubated at 37 °C for the desired period of time (0.5 to 3 h). The cells were extracted with 400 μl of lysis buffer (Cell Signaling Technologies) containing protein phosphatase and protease inhibitors. After 15 min on ice, insoluble material was pelleted by centrifugation (14,000× g) in a microfuge. The soluble supernatants were removed and frozen in aliquots at −80 °C. Phospho-CREB (pSer-133) was determined by western blotting as described in the previous section. β-Actin was used as a control for protein loading and normalization of phospho-CREB in these experiments because the intensity of signals from the pan (Total) CREB antibody was too weak for reliable measurement. ECL films were scanned with a flatbed scanner (Hewlett Packard, Palo Alto, CA) and and saved as TIF files. Images were imported into NIH Image J and band area intensities determined. A blank area of the blot was used for background subtraction. Images from three different samples from each population (AA, CA) were used for quantitation.

### Statistical analysis

Numerical data were expressed as mean±SEM. Data were analyzed by two-way ANOVA and Bonferroni posttests for statistical significance between groups (AA versus CA). One-way ANOVA analysis with Tukey’s pairwise comparisons were done for individual group column (time-based) data. Significance was based upon p<0.05. GraphPad Prism 4 was used for statistical analysis.

## Results

### Hydrostatic pressure-sensitive cAMP and CREB phosphorylation in ONH astrocytes

**Figure 1:**
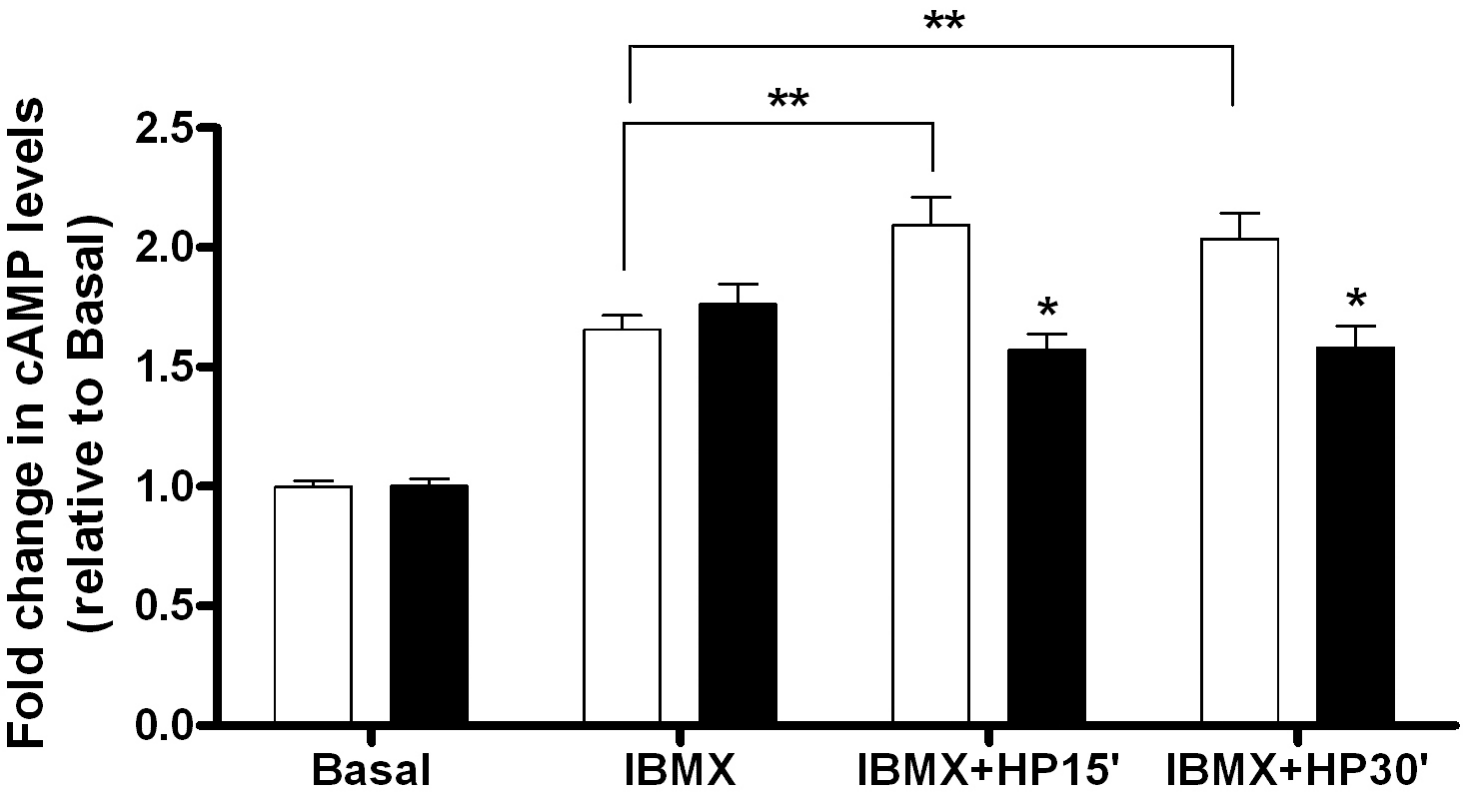
Sensitivity of cAMP levels to increased hydrostatic pressure in ONH astrocytes. In both AA (white bars) and CA (black bars) astrocytes, 500 μM IBMX stimulated cAMP, but pressure induced additional cAMP only in AA astrocytes. The basal cAMP levels in AA (59.6 pmol/mg protein) and CA (61.6 pmol/mg protein) astrocytes are not significantly different. The change in cAMP levels in AA astrocytes due to pressure stimulation is significant (p<0.01, double asterisk, n=8), and the difference between AA and CA astrocytes in terms of cAMP produced at 15 and 30 min of hydrostatic pressure is also significant (p<0.05, asterisk, n=8). Error bars indicate standard error of the mean.

**Figure 2:**
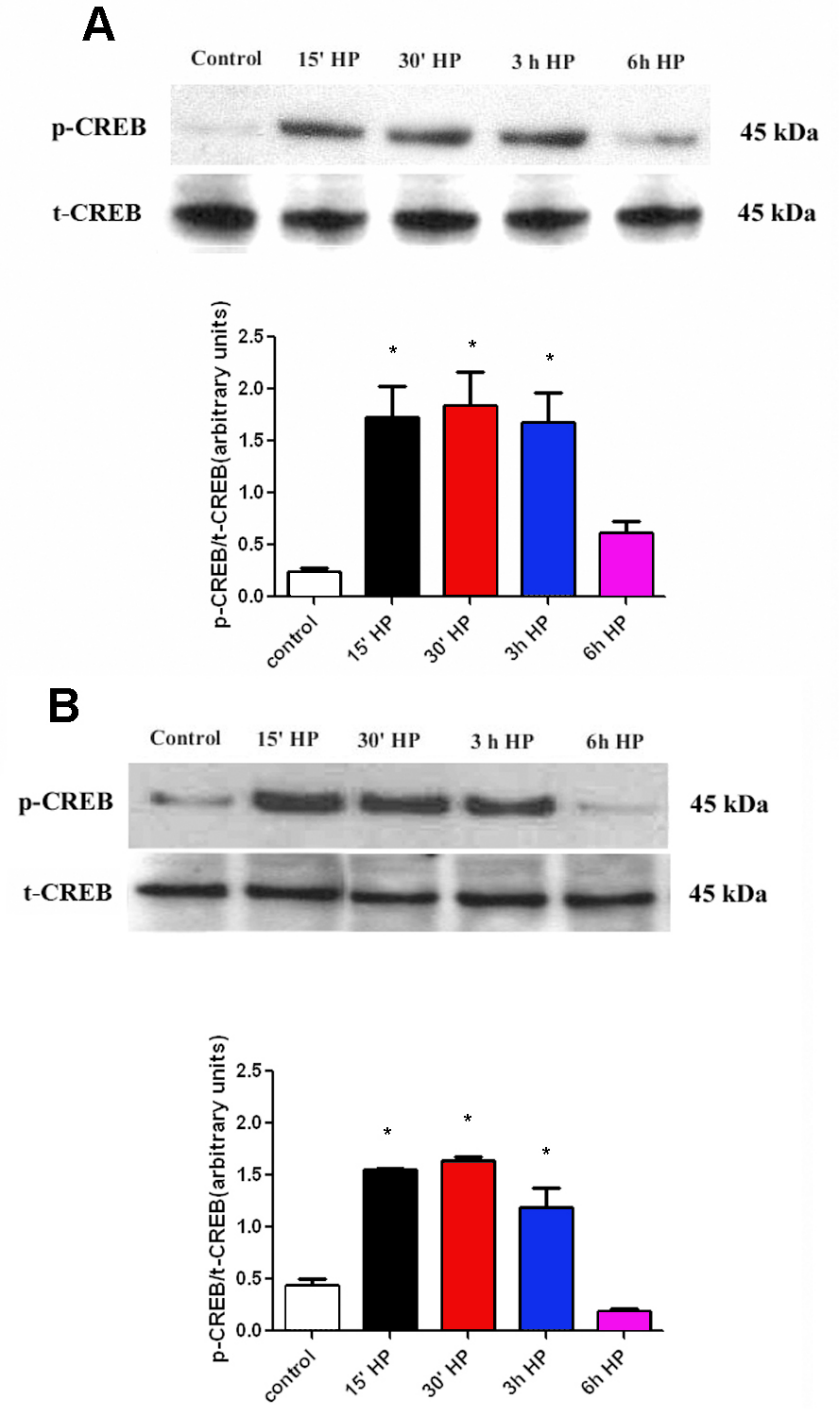
CREB phosphorylation is increased by hydrostatic pressure in ONH astrocytes. **A**: This panel contains a representative western blot of p-CREB (Ser-133) phosphorylation induced by elevated pressure in AA astrocytes over a 6 h period. **B**: Shown is a respresentative western blot of p-CREB phosphorylation induced by elevated pressure in CA astrocytes over a 6 h period. The bar graphs compare the data normalized to the total CREB protein in the extract. The basal levels of CREB are lower in AA astrocytes compared to CA astrocytes (p<0.05; asterisk, n=3). The fold-change of p-CREB in the AA astrocytes is higher than in the CA astrocytes (p<0.05; asterisk, n=3). Error bars indicate SEM.

**Figure 3:**
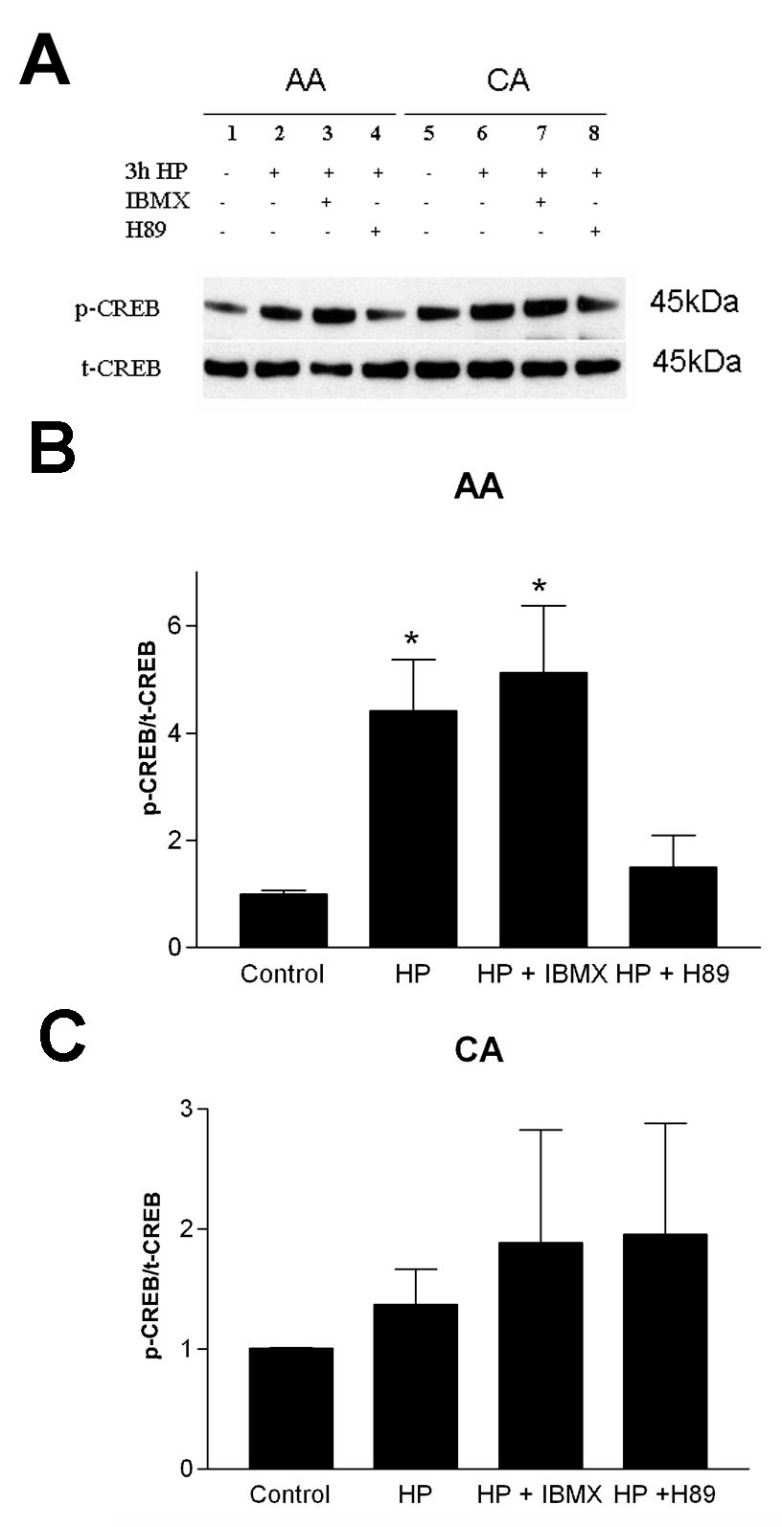
Hydrostatic pressure-induced phosphorylation of CREB is inhibited by H-89 in AA astrocytes. **A**: Shown is a representative western blot of p-CREB (Ser-133) phosphorylation and total CREB (t-CREB) in AA and CA astrocytes treated with 3h of elevated HP, IBMX, and HP + H89 (PKA inhibitor). Treatments do not alter total CREB in either cell type, but p-CREB phosphorylation is inhibited by H89 only in AA astrocytes. **B**: Bar graph of p-CREB/t-CREB (normalized to control=1.0) under various conditions for AA astrocytes (n=4) and panel **C**. CA astrocytes (n=3). Error bars indicate SEM. The change in p-CREB is significant (p<0.05, asterisk, n=3) in astrocytes treated with either pressure or IBMX.

Stimulation of cultured astrocytes for 15 min with phosphodiesterase inhibitor IBMX increased the cAMP levels in astrocytes. After exposure to elevated HP for 15 min and 30 min in the presence of IBMX, the intracellular cAMP levels further increased in AA astrocytes (p<0.01) compared to the basal levels. In contrast, cAMP levels of CA astrocytes were less affected by HP (Figure 1). The combined treatment (IBMX with HP) of CA astrocytes resulted in cAMP levels that were significantly lower than the AA cells (p<0.01). These data suggested that cAMP synthesis in AA astrocytes was more responsive to pressure. To determine whether changes in cAMP were reflected in the phosphorylation of a known substrate of cAMP-dependent PKA, we used endogenous CREB as a reporter for PKA activation. Although variable among individual donor astrocytes, the basal phosphorylation of CREB (p-CREB) tended to be lower in AA than in CA astrocytes (p<0.05). HP increased the phosphorylation of CREB (p-CREB) in 15 min and sustained it over a 3 h period (Figure 2). The change in p-CREB was 5–6 fold in AA astrocytes (Figure 2A) compared to a 2–3 fold change (Figure 2B) in CA astrocytes subjected to HP. To determine whether the phosphorylation was dependent upon PKA, we performed experiments in the presence of PKA inhibitor H-89. In AA astrocytes, pressure induced a significant (p<0.05) increase in p-CREB that was inhibited by PKA inhibitor H-89 (Figure 3A,B). Yet, p-CREB levels in CA astrocytes were less responsive to pressure treatment for 3 h and less responsive to inhibition by H-89 (Figure 3A,C).

### Ca^2+^ signaling and CREB phosphorylation in ONH astrocytes

**Figure 4:**
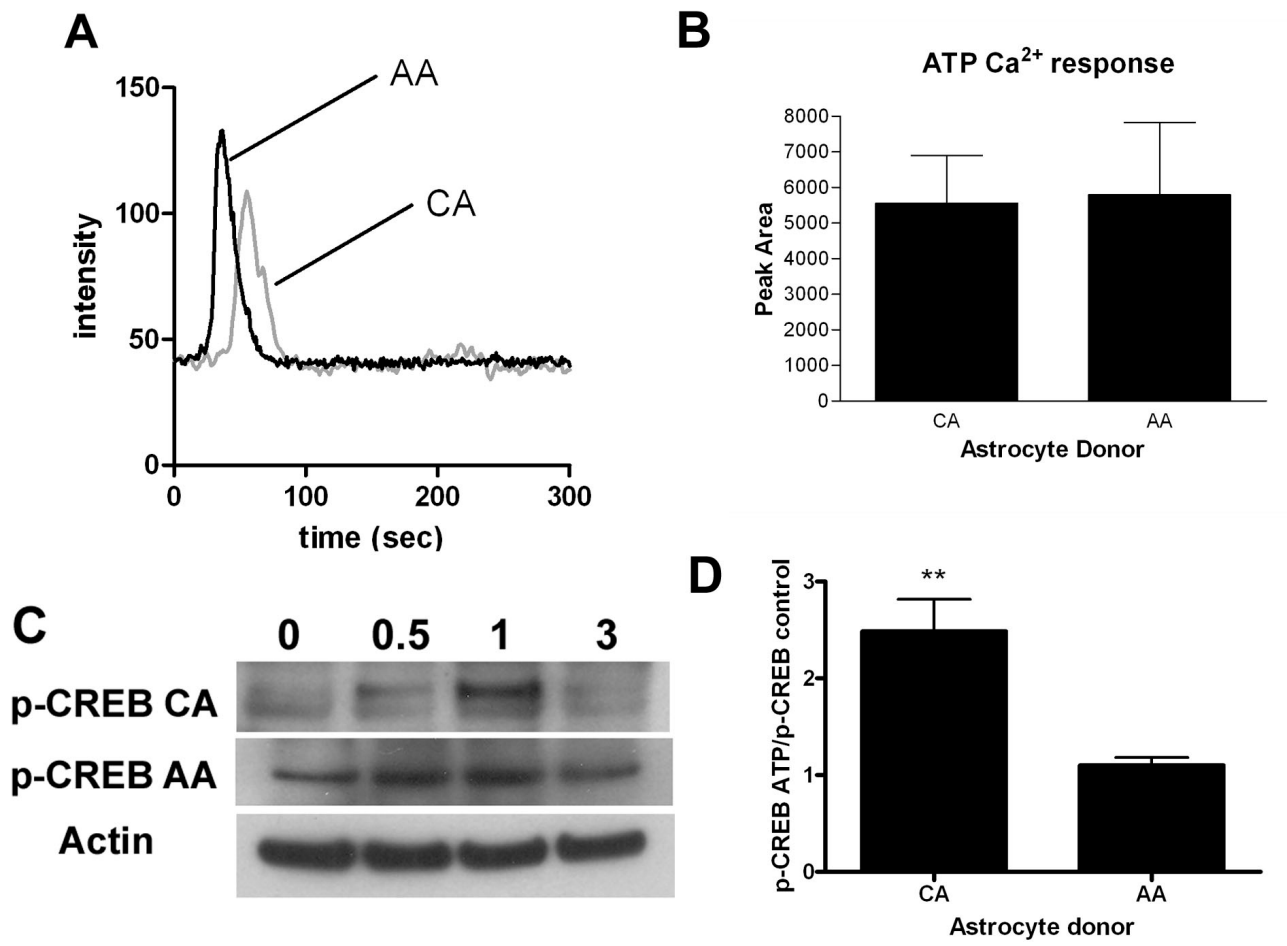
Effects of ATP on ONH astrocytes. **A**: Ca^2+^ traces were initiated by 1 mM ATP in ONH astrocytes. Cells were conditioned in serum-free medium containing Ca^2+^. Removal of extracellular Ca^2+^ had no effect on the transients, suggesting that ATP was acting at receptors coupled to G-protein mediated release of Ca^2+^ from intracellular stores. AA and CA astrocytes gave comparable responses. **B**: Peak areas were calculated from transients obtained from multiple cells (10–15) from three different donor lines. There was no significant difference in peak areas. **C**: Shown in this panel are representative western blots of p-CREB (Ser-133) phosphorylation in AA and CA astrocytes after treatment with ATP for 0, 0.5, 1, and 3 h. **D**: ATP stimulated more CREB phosphorylation in CA astrocytes compared to AA astrocytes treated for 1 h (p<0.01, double asterisk, n=3). Error bars indicate SEM.

Several kinases are known to be able to phosphorylate CREB [12]. For example, epidermal growth factor (EGF) signaling is known to activate CREB through the ERK-1 pathway that leads phosphorylation of CREB by the RSK2 kinase [16,17]. We have reported that HP activates EGF receptor signaling in ONH astrocytes [8]. However, when we tested various mitogen-activated protein kinase (MAPK; ERK-1, and p38) inhibitors, using a 10 μM dose, we observed no effect on p-CREB levels in either CA or AA astrocytes (not shown). Ca^2+^ signaling through Ca^2+^-dependent kinases also leads to CREB phosphorylation [12]. However, little is known about Ca^2+^ mobilization in ONH astrocytes. ATP is one of the known stimulators of Ca^2+^ mobilization in astrocytes [18]. Therefore, we investigated Ca^2+^ mobilization in ONH astrocytes stimulated by ATP and we looked at whether this stimulation is enough for CREB phosphorylation. Figure 4A illustrates that ATP does induce Ca^2+^ transients in ONH astrocytes measured using the fluorescent dye Fluo-4 [19]. The peak response of individual cells as well those cultured from different donors was quite variable, but there was no significant difference in the integrated areas of the Ca^2+^ response between AA and CA astrocytes (Figure 4B, n=6). Treating CA astrocytes with 100 μM ATP stimulated the phosphorylation of CREB with a peak at 1 h (Figure 4C). However, AA ONH astrocytes were somewhat less responsive to ATP stimulation of p-CREB as shown in Figure 4D. The average increase in p-CREB in CA astrocytes was 2.8 fold higher than control at 1 h, compared to a 1.1 fold change in the AA cells (Figure 4D). We found no change in p-CREB in AA astrocytes at the same time points. The difference in p-CREB change between CA and AA astrocytes was significant (p<0.01, n=3).

### Effects of pressure on cyclic AMP pathway genes in ONH astrocytes

**Figure 5:**
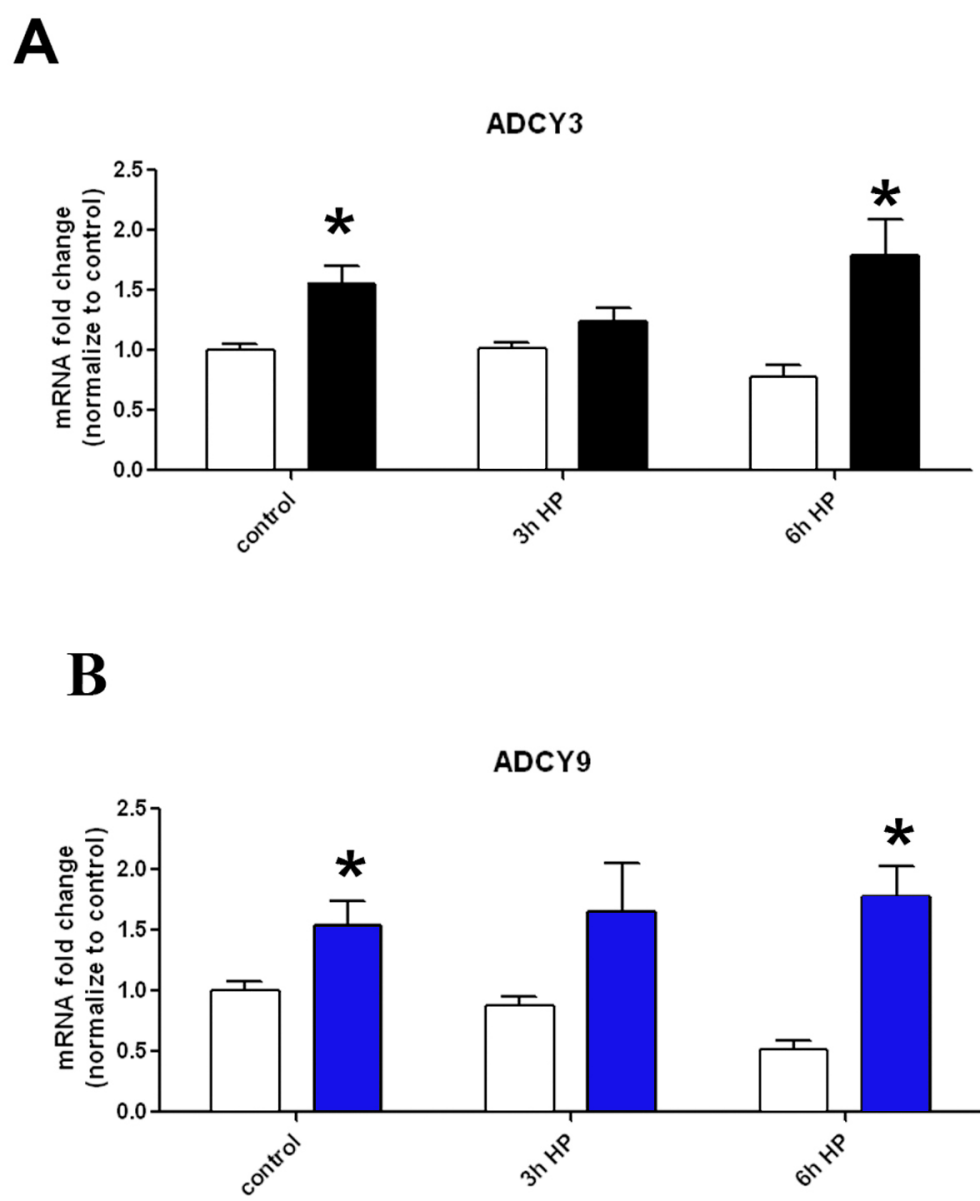
Effect of HP on the expression of adenylate cyclase mRNA in human ONH astrocytes. **A**: ADCY3 is upregulated in AA (black bars) compared to CA (white bars) astrocytes in the absence of HP (p<0.05, asterisks). Treatment with HP for 6 h also induces differential expression in AA compared to CA astrocytes (p<0.05, asterisk). **B**: Similar to ADCY3, ADCY9 is upregulated in AA (blue bars) compared to CA (white bars) astrocytes in the absence of HP (p<0.05, asterisk). Treatment with HP for 6 h also induces differential expression in AA compared to CA astrocytes (p<0.05, asterisk). In these experiments n=8 for controls and n=3 for the HP-treated groups. Error bars indicate SEM.

We used quantitative RT–PCR to determine the mRNA levels of two adenylate cyclase (ADCY) genes ADCY3 and ADCY9 in AA compared to CA astrocytes (Figure 5). Without stimulation, the basal levels of ADCY3 and ADCY9 were 1.7 fold higher in AA astrocytes compared to CA (p<0.001). After exposure to HP for 6 h, mRNA levels of ADCY3 and ADCY9 remained elevated in AA astrocytes (Figure 5A,B) compared to CA, however, the change in ADCY3 expression with respect to pressure within each group was not significant. ADCY3 after 6 h of HP treatment was 2.3 fold higher in AA astrocytes compared to the CA cells (p<0.001; Figure 5B). The ADCY9 mRNA level at 6 h of HP treatment was 48% lower in CA cells compared to control (p<0.05) while at 3 h the change was not significant. Between groups, ADCY9 was 3.5 fold higher in AA compared to CA after 6 h of HP (p<0.01).

**Figure 6:**
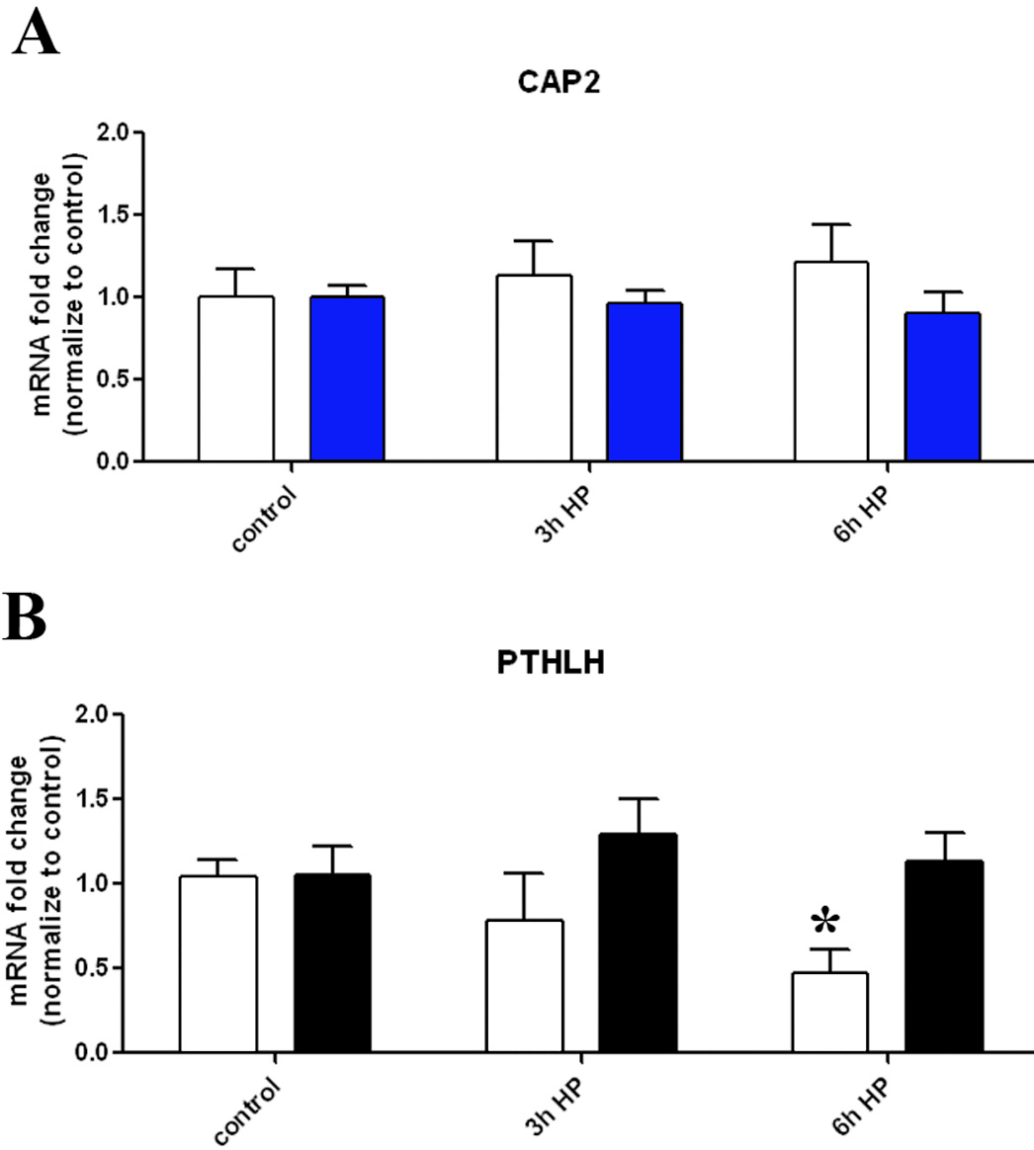
Effect of HP on CAP2 and PTHLH mRNAs in human ONH astrocytes. **A**: CAP2 expression is not affected by HP (3–6 h) in either AA (blue bars) or CA (white bars) astrocytes. **B**: PTHLH expression is higher in AA astrocytes (black bars) compared to CA astrocytes (white bars) after treatment with HP for 6 h (p<0.05, asterisk). Expression was determined using quantitative RT–PCR. Expression within each group was not significantly affected by 3–6 h of HP treatment. In these experiments n=8 for controls and n=3 for the HP-treated groups. Error bars indicate SEM.

The adenylate cyclase associated protein CAP2 enhances its activation [20]. Quantitative RT–PCR showed similar mRNA levels of CAP2 in either untreated or HP-treated AA and CA astrocytes (Figure 6A). Therefore, not all genes involved in regulating cAMP production were affected by HP. However, 6 h of HP treatment resulted in mRNA levels of parathyroid hormone-like hormone (PTHLH) that were 2.4-fold higher in AA astrocytes compared to CA astrocytes (Figure 6B; p<0.05). This appears to be primary a decrease in PTHLH levels in the CA cells at 6 h of HP treatment. PTHLH activates cAMP synthesis and signaling through ubiquitous PTH receptors [21,22]. PTHLH has cAMP regulatory elements in its promoter [23], providing a putative positive feedback mechanism for increasing cAMP.

**Figure 7:**
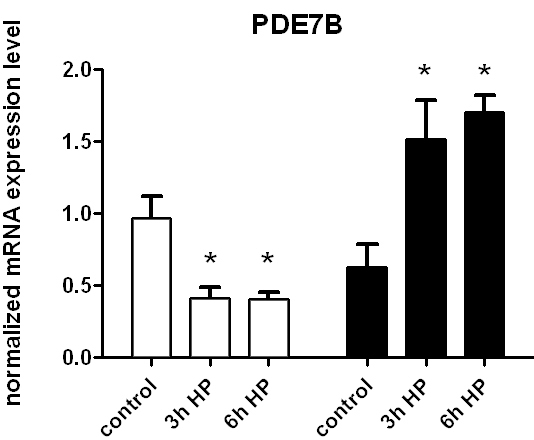
Effects of HP on the expression of PDE7B mRNA in human ONH astrocytes. PDE7B expression is significantly (p<0.05, asterisk) increased in AA astrocytes (black bars) compared to control after treatment with HP for 3 or 6 h. On the other hand, HP treatment significantly (p<0.05 asterisk) decreases expression of PDE7B in CA astrocytes (white bars) compared to control after treatment with HP for 3 or 6 h. In these experiments n=8 for controls and n=3 for the HP-treated groups. Error bars indicate SEM.

Finally, the differential modulation of CREB phosphorylation in AA vs CA astrocytes under pressure caused altered expression of another known cAMP-regulated gene, the cAMP-specific phosphodiesterase, PDE7B. Under basal conditions, PDE7B mRNA was not significantly different between AA and CA astrocytes. However, PDE7B mRNA was downregulated by pressure in CA astrocytes by HP treatment for 3 or 6 h and upregulated in AA astrocytes resulting in a 3.7–4.2 fold difference between these groups over time (p<0.05; Figure 7). This results underscores that there must be differences in CREB-mediated transcriptional events between AA and CA astrocytes.

## Discussion

Intracellular cAMP levels in unstimulated astrocytes were similar in AA and CA astrocytes (Figure 1), but in AA astrocytes cAMP synthesis appeared to be more sensitive to hydrostatic pressure. Thus, the enhanced sensitivity of AA astrocytes is unique to this population. There is an earlier report on the stimulation of cAMP production in cultured endothelial cells derived from Schlemm’s canal [24], however, this is the first report of a pressure effect on cAMP in ONH cells. We found that pressure-induced CREB phosphorylation is dependent upon PKA in AA astrocytes because it was inhibited by PKA inhibitor H-89.

Both ADCY3 and ADCY9 are activated by G-protein (Gs) stimuli and further regulated through Ca^2+^-dependent processes. ADCY3 activity is decreased through Ca^2+^-calmodulin protein kinase (CAMKII) phosphorylation [25]. Alternatively, ADCY9 is stimulated by CAMKII phosphorylation [26]. The parathyroid hormone receptor ligand, PTHLH, is differentially regulated in AA compared to CA astrocytes by HP treatment, providing a means by which adenylate cyclase activity may be stimulated in these cells in an autocrine fashion. These findings are consistent with our recently published results, which show an increase of PTHLH expression in glaucomatous astrocytes [5]. We should point out, however, that changes in gene expression may not be reflected at the protein level, especially during HP treatments; we have not yet determined whether there are changes in protein turnover via proteosomal, or lysosomal processes. PDE7B, a cAMP selective phosphodiesterase is also differentially upregulated in AA compared to CA astrocytes by increased HP (Figure 7). Thus, the upregulation of PDE7B may serve as one of the sources of negative feedback for cAMP production in AA ONH astrocytes.

CA astrocyte p-CREB levels may be modulated through both cAMP and Ca^2+^-dependent pathways, because we found that ATP, which mobilizes Ca^2+^ from internal stores, results in increased CREB phosphorylation in CA astrocytes, while AA astrocytes are less responsive. ATP is secreted from glia cells in the retina by mechanical stimulation [27] and by HP changes [28,29] causing increased ATP secretion. Thus, activation of the cAMP-dependent signaling pathways and ATP secretory pathways may both contribute to increased susceptibility to elevated intraocular pressure-related stress in ONH astrocytes. The time delay (about 1 h) between the Ca^2+^ transient elicited by ATP and the observed increase in CREB phosphorylation (Ser-133) is consistent with previous observations in other types of astrocytes [18]. We suspect that this might involve a cascade of events that leads to the translocation of CREB and other transcriptional factors to the nucleus for activation. CREB phosphorylation, however, does not necessarily lead to changes in gene transcription in astrocytes [30]. Therefore, the differences we observe in gene expression between the AA and CA populations of astrocytes likely involves additional transcriptional regulators. Similarly, the current work as well as previously published studies of changes in gene expression after longer intervals of HP treatment (24–48 h, e.g., [8,10]) indicates that early signaling changes are just the first step in a process that enables ONH astrocytes to transition into the “reactive” phenotype found in glaucoma. Further studies are needed to elucidate the interaction of the cAMP and other signaling pathways involved during the development of optic nerve neuropathy of glaucoma.
